# Hospital News

**Published:** 1922-06

**Authors:** 


### HOSPITAL NEWS.

The new Victory Wing at the Devon and Exeter Hospital will be opened on June 20 by Viscount Hambleden.

It has been decided to add the word " Chelsea " to the title of the Kensington and Fulham General Hospital.

After the June quarter the Brighton Hospital for Women expects to move into its new building in Buckingham Road.

It is reported that the Women's Hospital for Children, Harrow Road, is being closed for want of funds.

Sir Edward Brotherton, M.P., has given a new X-ray apparatus to Clayton Hospital, Wakefield.

The method of treatment by X-rays in use at Erlangen, Bavaria, has been adopted with the necessary equipment at the Birmingham and Midland Hospital for Skin and Urinary Diseases.

A new X-ray department and pathological laboratory has been presented to Lincoln County Hospital by the Lincolnshire Yeomanry, as a memorial of the officers and men who fell in the war.

Dr. Harwood Nutt, a radiologist, for many years on the staff of the Royal Hospital, Sheffield, who has suffered injury from X-rays, and is temporarily incapacitated, has been presented with a four-figure cheque by his professional and other friends.

The City of London Hospital for Diseases of the Chest, Victoria Park, contemplates increasing the number of beds allotted to heart and non-tubercular lung cases. The income derived from payments made by public authorities for tuberculous cases is being reduced.

A special orthopædic department, with fifty beds, is being formed at Leeds General Infirmary, where the treatment of all the categories of this work will be concentrated. This will eventually necessitate extension when the necessary funds are available.

The Presbyterian Health Insurance Society has opened a Rest Home for members at Portrush, Ireland, in the building formerly known as the Hotel Metropole. The Rest Home can accommodate twenty patients, ten men and ten women, and has been provided out of a surplus in the Society's hands.

Mr. Thomas Thornton, the night porter at the Belgrave Hospital for Children, lately made a plucky attempt to capture a midnight burglar who attacked him with a knife. Though the man escaped, he secured nothing. Indeed, he left behind an overcoat and boots, which it is suggested should be sold for the hospital's funds. Mr. Thornton was formerly in the Coldstream Guards.

The troublous times in Ireland are reflected in the report of the Royal Victoria Hospital, Belfast, where a considerable number of gun-shot wounds have been treated. On several occasions the accommodation has been taxed to the utmost to provide for all the casualties seeking admission. This has necessitated the postponement of cases otherwise placed on the urgent list, and a women's ward has had to be appropriated for male surgical cases.

Since the wards were opened, sixty-five patients have been received at the London Jewish Hospital. The extension of the out-patient department is contemplated, and the ground at the back of the institution is being laid out as a garden for patients and staff. When the surgical wards are in use, the Nurses' Home will have to be extended. The lease of the present home was bought in 1920, and that it may be extended, the freehold has now been purchased. The X-ray department is being equipped at a cost of £800.

The remaining " mystery tower " at Shoreham is being opened to the public, and the proceeds of admission will be given to the Royal Sussex County Hospital, Brighton.

A penalty clause is reported to have been adopted by the Croydon Guardians because of the number of probationers who break their contracts.

A hospital for the treatment of surgical tuberculosis in children is being opened at Heatherwood, Ascot, by the United Services Fund.

A piece of plate has been presented by the Western Infirmary, Glasgow, to Lord Glenarthur, who was its chairman for seventeen years. He was for thirty-four years a member of the Board of Management.

The South African Hospital, housed in huts in Richmond Park, is being used for the neuropathic cases hitherto at Tooting. It is objected that the huts should now be removed, and an institution acquired elsewhere.

The Prince of Wales' Hospital, Cardiff, where limbless soldiers and sailors were treated, extended its work to cripples in 1921. It is now open for the treatment of adults and children, no less than for the fitting of artificial limbs and surgical appliances.

The Jessop Hospital, Sheffield, has extended the radius visited by its midwives to one mile and a half from the hospital, so that many more patients are eligible for maternity treatment in their own homes. A lady almoner is being considered.

The employees of the Newcastle Corporation Tramways have named a cot in the Charlotte E. A. Stephenson Ward of the Philipson Children's Sanatorium, at Stannington, Northumberland, to which their medical charities fund has sent a donation for several years.

Some friends of the South London Hospital for Women have presented it with the freehold of a large adjoining house. Their gift will provide thirty more beds for surgical cases, together with quarters for the staff. The urgent need for extension is temporarily lessened.

On the roof of the Queen's Hospital for Children, Hackney, a series of enclosed " Wardlets " is contemplated. Each child will have one, which a glass partition will separate from a gangway. The idea is that babies thrive better apart, and the proposed plan will enable them to be easily watched while fully isolated.

The recent death of Dr. Hugh E. Fraser removed a hospital administrator who had held the post of medical superintendent of Dundee Royal Infirmary for twenty-five years Educated at Edinburgh, where he took the M.D. in 1897 and received many prizes, Dr. Fraser held posts in the Edinburgh Royal Maternity Hospital and the Northern Infirmary Inverness. He had several extra-professional interests, and was a Fellow of the Society of Antiquaries.

The German Hospital, Dalston, which is one of those hospitals where beds have had to be closed, has made an interesting departure. Of the thirty closed beds twelve have been reopened and reserved for aged Germans suffering from incurable diseases. Six men and six women have thus been saved from the workhouse infirmary. Private patients are also treated in the sanatorium. The Hospital Saturday Fund has resumed its contributions, but no grant has lately been received from King Edward's Hospital Fund. The patients, of course, include many English, and last year in and out-patients contributed about £4,000 to the hospital. The number of patients treated continues to grow. The ordinary income last year was £17,000, or £1,800 short of the expenditure. All things considered, the hospital is doing well. It has seventy-seven years' work behind it.

The Royal Bath Hospital, Harrogate, has reopened after the improvements made during the winter. A new Vichy douche, Berthollet and paraffin wax baths have been added; the nurses' quarters and heating plant have been remodelled; and bacteriological, dental, and electrical laboratories have been formed for the investigation of rheumatism and allied conditions.

Dr. A. Bertram Soltau has declared that he and his colleagues on the honorary staff of the South Devon and East Cornwall Hospital do not accept the British Medical Association Report (which we summarised last month), and reject its idea that a fixed percentage of the hospital income should be paid to hospital staffs. They preferred to maintain their honorary status.

Miss Mary F. Bostock, R.R.C., Lady Superintendent of the Royal Victoria Hospital, Belfast, has retired after an association of thirty-five years. For fifteen years she was matron of the Throne Hospital, the children's branch, and then at the Royal, the new buildings of which were opened by King Edward in 1903. Hers is a long and remarkable record of experience, the more perhaps for being associated with one institution in one place.

The principle of amalgamation between the Norfolk and Norwich Hospital and the Norfolk and Norwich Eye Infirmary has been agreed. The identity of the Eye Infirmary is to be retained, and a combined appeal would precede the building of any ophthalmic block at the hospital. If the joint committee in charge of the details comes to a favourable decision, the head-quarters of the Eye Infirmary will be removed to the Hospital, and the house now occupied by the Eye Infirmary be used for the additional accommodation of nurses.

At the annual Easter banquet at the Mansion House on April 26, the Lord Mayor, proposing " Prosperity to the Royal Hospitals," said that in 1552-3 the citizens of London laid the foundation of the hospitals which Henry VIII. had handed over to them. Only on the previous day the Lady Mayoress had taken part in a little ceremony which commenced the demolition of the old nurses' quarters of St. Bartholomew's Hospital. In their place was to be erected a modern block. The hospital was able to do that owing to the munificence of Sir Edward Stern, who had given £25,000, and of Sir John Bell, who had contributed £10,000. Eight hundred years had passed since St. Bartholomew's was founded, and to-day it was doing as splendid a work as ever. St. Thomas's was another City hospital, and he regretted that it was suffering at the moment from lack of funds, and had a number of wards closed.

---

The Voluntary Hospitals Commission has considered the preparation of a simplified uniform system of accounts which could be recommended for adoption by hospitals to which the uniform system of King Edward's Fund may be inapplicable. The Commission has appointed a small Sub-Committee to draft proposals for this purpose, and has secured the assistance of Captain H. G. Howitt, D.S.O., of Messrs. W. B. Peat & Co., who helped to prepare the financial statements for Lord Cave's Committee on the Voluntary Hospitals.

---

" Shell Brand " Floor Polish was the first polish ever offered to public institutions. It is still the best, because a very little " Shell Brand " goes a long way, and a few light rubs creates a brilliant polished surface which lasts. We have found it, moreover, an admirable furniture polish, and it gives to linoleum a pleasing surface which is not unduly slippery. The institutions which have tried " Shell Brand " have continued to use it; but there are still some who have not yet given this excellent polish a test. Messrs. Hamilton & Co. Ltd., are always pleased to send a sample for testing to anyone who writes to 6, Possilpark, Glasgow.

### Other News.

The journal of the Charity Organisation Society, which recently died, has come to life again in the form of a " Quarterly," price 4d.

Mr. T. R. Thornton, speaking before the congress of the Eastern Osteopathic Society of the United States, declared that the Venus de Milo has " a neurasthenic stomach which is not in the proper position."

The Rockefeller Foundation has offered five scholarships of one thousand dollars each, plus all expenses, for Indian medical graduates to take post-graduate courses in America in public health. The selection will be made by the Scientific Board of the Indian Research Fund.

According to the list issued by the General Medical Council the number of medical and dental students fell from 3,240 in 1919 and 2,531 in 1920 to 1,808 last year. The reduced number is accounted for by the cessation of the Government grant to ex-Service students. The average number of students for the five years before the war was 1,400.

Professor W. S. Lazarus-Barlow, Professor of Experimental Pathology, and Director of Cancer Research laboratories at the Middlesex Hospital Medical School, has just returned from a six weeks' tour in the United States, where he has been investigating the most recent methods of cancer research and radium treatment.

In response to the request of the Seamen's Institute of New York, the Washington public health service is prepared to give medical advice by wireless in language intelligible to the layman to doctorless ships calling for aid. Vessels without surgeons will be equipped with hearing-sets. A New York hospital reports the arrival at 10 o'clock at night of a message from a steamship saying that a man on board was suffering from pain in the abdomen, persistent vomiting, and inability to lie down owing to the pain. The officer of the day promptly prescribed treatment; and the next morning the ship wirelessed thanks, saying that the patient was much improved.

The London Committee of the Cancer Research Fund, Ireland, hopes to establish a cancer research department in connection with the Skin and Cancer Hospital, Hume-street, Dublin. It has decided to issue a pamphlet setting forth the aims of the committee, to arrange a flag day on behalf of the fund, to ask the heads of religious denominations to institute a " Cancer Sunday " each year, and to appeal to the Medical Graduates' Association for support. Ireland has no institution to deal specially with research, and only one hospital giving specialist treatment to cancer and lupus patients. Competent authorities state that cancer in that country is overtaking consumption in the number of its victims, and that one out of every ten persons over forty years of age dies from that disease.

The Wounded Soldiers' Drives and Recreation Fund, working under the Joint Council of the Order of St. John and the British Red Cross Society, arranged during 1921, drives, river trips, concerts, theatrical and other parties at which 22,848 men were entertained; concerts at the Orpington Hospital during November last were attended by 2,530 patients, and thanks to the Eccentric Club, the Not-Forgotten Association and several amateur dramatic societies, further entertainments were arranged, bringing the number of men entertained to 35,000. The funds for this commendable work are supplied by the Joint Council; the expenses of administration, apart from what has been spent on the men, have amounted to only 2s. 8d. during the year. There are 5,000 men in the hospitals in and around London, and an urgent need still exists for offers from hosts who are willing to entertain these men in parties ranging from three or four to 100. Such offers should be sent to Mr. F. S. Phillips, O.B.E., 27 Grosvenor Place, S.W., who will make all the necessary arrangements free of trouble or cost to the hosts.

